# Prescriptions patterns and appropriateness of usage of antibiotics in small and medium- sized hospitals in Korea

**DOI:** 10.1017/ash.2022.88

**Published:** 2022-05-16

**Authors:** Yong Chan Kim, Ji Young Park, Bongyoung Kim, Eu Suk Kim, Hyuk Ga, Rangmi Myung, Se Yoon Park, Myung Jin Lee, Song Mi Moon, Sun Hee Park, Kyoung-Ho Song, Hong Bin Kim

## Abstract

**Background:** Although small- and medium-sized hospitals comprise most healthcare providers in South Korea, data on antibiotic usage is limited in these facilities. We evaluated the pattern of antibiotic usage and its appropriateness in hospitals with <400 beds in South Korea. **Methods:** A multicenter retrospective study was conducted in 10 hospitals (6 long-term care hospitals, 3 acute-care hospitals, and 1 orthopedic hospital), with <400 beds in South Korea. We analyzed patterns of antibiotic prescription and their appropriateness in the participating hospitals. Data on the monthly antibiotic prescriptions and patient days for hospitalized patients were collected using electronic databases from each hospital. To avoid the effect of the COVID-19 pandemic, data were collected from January to December 2019. For the evaluation of the appropriateness of the prescription, 25 patients under antibiotic therapy were randomly selected at each hospital over 2 separate periods. Due to the heterogeneity of their characteristics, the orthopedics hospital was excluded from the analysis. The collected data were reviewed, and the appropriateness of antibiotic prescriptions was evaluated by 5 specialists in infectious diseases (adult and pediatric). Data from 2 hospitals were assigned to each specialist. The appropriateness of antibiotic prescriptions was evaluated from 3 aspects: route of administration, dose, and class. If the 3 aspects were ‘optimal,’ the prescription was considered ‘optimal.’ If only the route was ‘optimal,’ and the dose and/or class was ‘suboptimal,’ but not ‘inappropriate,’ it was considered ‘suboptimal.’ If even 1 aspect was ‘inappropriate,’ it was classified as ‘inappropriate.’ **Results:** The most commonly prescribed antibiotics in long-term care hospitals was fluoroquinolone, followed by β-lactam/β-lactamase inhibitor (antipseudomonal). In acute-care hospitals, these were third-generation cephalosporin, followed by first-generation cephalosporin and second-generation cephalosporin. The major antibiotics that were prescribed in the orthopedics hospital was first-generation cephalosporin. Only 2.3% of the antibiotics were administered inappropriately. In comparison, 15.3% of patients were prescribed an inappropriate dose. The proportion of inappropriate antibiotic prescriptions was 30.6% of the total antibiotic prescriptions. **Conclusions:** The antibiotic usage patterns vary between small- and medium-sized hospitals in South Korea. The proportion of inappropriate prescriptions exceeded 30% of the total antibiotic prescriptions.

**Funding:** None

**Disclosures:** None

